# Supplementary material for: Changes in orogenic style and surface environment recorded in Paleoproterozoic foreland successions
Source: Nat Commun. 2023 Dec 2;14:7997. doi: 10.1038/s41467-023-43893-w (PMC10693560; doi:10.1038/s41467-023-43893-w)
Supplement: Supplementary file 1 — Supplementary Information [file 41467_2023_43893_MOESM1_ESM.pdf]

Supplementary Information for

Changes in orogenic style and surficial environment recorded in Paleoproterozoic foreland successions

Bo Huang*, Man Liu, Timothy Kusky, Tim E. Johnson, Simon A. Wilde, Dong Fu, Hao Deng, Qunye Qian

*Corresponding author. Email: bohuang@cug.edu.cn (B.H.)

The supplementary information includes:

Supplementary Note 1: Geological background of the North China Craton

Supplementary Note 2: Zircon and rutile U–Pb geochronology

Supplementary Note 3: $^{40}Ar/^{39}Ar$ geochronology

Supplementary Note 4: Estimation of metamorphic *P*–*T* conditions

Supplementary Figures 1 to 11

Supplementary Tables 1 to 3

Supplementary Note

1. Geological background of the North China Craton

The North China Craton (NCC) records long-term crustal growth and reworking with the oldest rocks up to ca. 3.8 Ga. It also preserves well-exposed rock assemblages formed during the late Archean and Paleoproterozoic transition, providing a natural laboratory to explore crustal growth, continental assembly, and their general implications for secular change in tectonic regimes[1-11]. During the past three decades, two major tectonic-magmatic-metamorphic events (ca. 2.55–2.48 Ga, and ca. 2.0–1.85 Ga) have been identified based on multiple lines of evidence, including isotopic geochronology, metamorphic *P*-*T*-*t* paths[3,12-17], magmatic assemblages[4,18-30], and structural styles[31-38], which have been linked with two stages of subduction-collision orogenesis[1,2] or cratonisation[6,7,39,40], and implying the operation of plate tectonics at least by the Neoarchean[41-43].

Nevertheless, several different tectonic models have been proposed to explain the formation of the basement of the NCC. Zhai et al.[6,7,39,44] suggested that several microcontinental blocks assembled along greenstone belts at the end of the Archean (first episode of cratonisation), which was followed by tectonic quiescence and rifting-subduction-collision along three Paleoproterozoic mobile belts. Zhao et al.[9-11] suggested a prolonged eastward subduction of paleo-oceanic lithosphere occurred between the Eastern and Western blocks since 2.56 Ga, which was terminated by collisional orogenesis at ca. 1.85 Ga. Kusky et al.[1,2,45] suggested that multiple stages of accretionary and collisional orogenic events occurred from ca. 2.5 Ga to 1.8 Ga, including eastward subduction, arc-continent collision (Central Orogenic Belt), subduction polarity reversal, accretion of oceanic plateau (Ordos block), development of an Andean-type continental margin and continental collision along the northern margin

of the NCC, which led to the outward growth of the NCC and final incorporation into the Columbia supercontinent[46,47]. Thus, the core debate focuses on 1) whether there was a late Archean collision between continental blocks or between arcs and microcontinents[44,45] or not[9], and 2) the nature of the early Paleoproterozoic evolutionary process from 2.5 Ga to 1.8 Ga[6,44]. In addition, some general and frontier scientific questions regarding the styles of orogenesis and plate tectonics require further constraints from different perspectives[43,48]. The sedimentary archives from orogenic forelands record erosion and sedimentation of detritus (including various magmatic and metamorphic minerals) from adjacent orogenic crust, thus providing an excellent window to solve the above-mentioned issues.

The Songshan Group unconformably overlies the Archean basement in the southern NCC and has been interpreted as either a post-cratonisation sedimentary succession developed along the cratonic or rift margin[39], a retro-arc foreland basin developed behind an Andean-type continental arc[40,49], or a peripheral to retro-arc foreland basin[2,31]. The latter two models similarly interpreted the succession as a result of the eastward subduction of oceanic lithosphere beneath the Proto-Eastern Block, although the depositional ages and deformational history of the Songshan Group remain incompletely constrained[49-51].

In this study, we focused on the deposition and deformation of the Songshan Group, and conducted detailed structural investigations and multi-mineral geochronological analysis. These datasets enable us to better constrain its depositional-to-deformational evolution and characterise the two orogenic styles in the NCC during the Neoarchean to Paleoproterozoic transition from sedimentary and deformational perspectives that have been largely underestimated in the past.

2. Zircon and rutile U–Pb geochronology and trace elements

A total of eight samples were selected for zircon U–Pb dating and four samples containing rutiles were selected for U–Pb dating (see Methods and Supplementary Data 1). The results of the zircon and rutile analyses and the maximum depositional ages are described below. Three age criteria, including YSG (youngest single grain), YC1σ (youngest cluster overlapping at 1σ uncertainties) and YC2σ (youngest cluster overlapping at 2σ uncertainties), were calculated and used to characterise the maximum depositional ages[52-54]. The MDA is listed in Supplementary Table S1.

2.1. Zircon

2.1.1. U–Pb geochronology

Sample 20SS10 is a quartz-mica schist of the Archean Dengfeng Complex that underlies the Songshan Group (Fig. 2). Most zircon grains are prismatic, with length/width ratios of 2:1–3:2, and exhibit oscillatory zoning in cathodoluminescence (CL) images (Supplementary Fig. 6a). Some grains have thin white rims. The analysed zircons have Th contents of 6–103 ppm and U contents of 19–154 ppm and Th/U ratios of 0.15–1.20. The ages range from 2709 Ma to 2528 Ma, with a single peak at 2.57 Ga (Supplementary Fig. 7b). The YSG, YC1σ and YC2σ provide ages of 2539 ± 35 Ma, 2560 ± 7 Ma (n=39) and 2573 ± 6 Ma (n=52), respectively.

Sample 20SS09 was collected from the relatively homogenous matrix of the thick metamorphosed basal conglomerate of the Luohandong Formation in the lowermost Songshan Group that unconformably overlies the Dengfeng Complex (Fig. 2). Zircon grains are prismatic to stubby, with lengths of ~80–150 μm and length/width ratios from 1:1 to 2:1. They exhibit well-developed

oscillatory zoning, with minor zircon grains (~12%, 14 out of total 121 grains within the zircon mount) containing thin bright rim in CL images (Supplementary Fig. 6b). Analyses have low to moderate concentrations of Th (7–198 ppm) and U (20–344 ppm), with Th/U ratios from 0.05 to 1.45. The ages range from 2889 Ma to 2429 Ma, with a single peak at 2.60 Ga (Supplementary Fig. 7d). The YSG, YC1σ and YC2σ record ages of 2429 ± 54 Ma, 2465 ± 22 Ma (n=5) and 2539 ± 10 Ma (n=19), respectively.

Sample 20SS01 was collected from a quartzite of the Luohandong Formation. Zircon grains are either fragmented or well-rounded, with lengths of ~100–150 μm and length/width ratios from 1:1 to 2:1. They mostly exhibit oscillatory to banded zoning, reflecting magmatic origins; while minor grains have thin bright rims (~9%, 20 of total 211 grains) (Supplementary Fig. 6c). All analyses have low to moderate concentrations of Th (5.7–154 ppm) and U (15–272 ppm), with Th/U ratios from 0.23 to 1.43. The zircon ages define two significant populations, with a diagnostic population clustering at 3.37 Ga (n=6) and another population clustering at 2.45–2.75 Ga (n=88). The YSG, YC1σ and YC2σ ages are 2377 ± 45 Ma, 2429 ± 19 Ma (n=4) and 2475 ± 8 Ma (n=19), respectively.

Sample 20SS07 was collected from another quartzite of the Luohandong Formation. The majority of zircon grains are ellipsoidal and well-rounded, with lengths of ~60–180 μm and length/width ratios from 1:1 to 3:1. Most grains exhibit oscillatory and banded zoning, except for ~8% (17 out of 212 grains) with thin rims (Supplementary Fig. 6d). Analyses range from low to high concentrations of Th (12–173 ppm) and U (25–955 ppm), with Th/U ratios from 0.10 to 1.90. The results are dominated by Neoarchean ages, with peaks at 2.63 Ga, 2.56 Ga, and 2.52 Ga. The YSG, YC1σ and YC2σ ages are 2488 ± 29 Ma, 2511 ± 10 Ma (n=13) and 2516 ± 10 Ma (n=10), respectively.

Sample 20SS05 is a quartzite from the Wuzhiling Formation. Zircon grains are prismatic to stubby, with lengths of ~50–140 μm and length/width ratios from 1:1 to 2:1. They exhibit strong oscillatory zoning, and minor grains have thin rims (~4%, 9 out of 220 grains) (Supplementary Fig. 6e). All analyses have low to moderate concentrations of Th (6–208 ppm) and U (27–267 ppm), with Th/U ratios from 0.23 to 1.32 (except for one with 0.03). The zircon ages show two main populations, with one clustering at 2.72 Ga and another at 2.49 Ga. The YSG, YC1σ and YC2σ ages are 2454 ± 42 Ma, 2490 ± 8 Ma (n=34) and 2512 ± 7 Ma (n=51), respectively.

Sample 20SS06 is a quartz-mica schist from the Wuzhiling Formation. Zircon grains are prismatic to stubby, with lengths of ~60–160 μm and length/width ratios from 1:1 to 2:1. They exhibit oscillatory or banded zoning, with minor grains having thin rims (~3%, 7 out of 212 grains) (Supplementary Fig. 6f). All analyses have low to moderate concentrations of Th (6–195 ppm) and U (18–298 ppm), with some relatively high Th/U ratios from 0.19 to 1.21. The analyses are dominated by Neoarchean ages, with a strong peak at 2.52 Ga. The YSG, YC1σ and YC2σ ages are 2477 ± 39 Ma, 2507 ± 6 Ma (n=35) and 2528 ± 4 Ma (n=59), respectively.

Sample 21SS25 is a quartzite from the Huayu Formation. Zircon grains are stubby and well-rounded, with lengths of ~60–150 μm and length/width ratios from 1:1 to 2:1. They exhibit oscillatory or broad banded zoning (Supplementary Fig. 6g). All analyses have moderate concentrations of Th (9–351 ppm) and U (26–232 ppm), with relatively high Th/U ratios from 0.33 to 1.99. The zircon ages define a main population clustering at 2.52 Ga, with subordinate but diagnostic Paleoproterozoic populations ranging in age from 2.21 Ga to 2.45 Ga. The YSG, YC1σ and YC2σ ages are 2218 ± 35 Ma, 2241 ± 20 Ma (n=4) and 2275 ± 15 Ma (n=7), respectively.

Sample 20DF03 is a quartz sandstone from the Maanshan Formation of the overlying Wufoshan Group. Zircon grains are ellipsoidal and well-rounded, with lengths of ~100–200 μm and length/width ratios from 1:1 to 2.5:1. They exhibit oscillatory, patchy or banded zonings, and some grains have core–rim structures with a proportion of ~21% (45 out of 212 grains, Supplementary Figs. 6h and 8a). Analyses have variable concentrations of Th (3–304 ppm) and U (26–362 ppm), with Th/U ratios from 0.02 to 1.97. The zircon ages range from 2.9 Ga to 1.79 Ga, with complex age patterns and several minor peaks at 2.67 Ga, 2.56 Ga, 2.45 Ga, 2.34 Ga, 2.14 Ga and 1.96 Ga. The YSG, YC1σ and YC2σ ages are 1798 ± 33 Ma, 1813 ± 11 Ma (n=13) and 1849 ± 7 Ma (n=24), respectively.

2.1.2. Trace element characteristics

The rare earth element (REE) compositions of zircon grains from the Dengfeng Complex and Songshan Group are distinct from those of the Wufoshan Group (Supplementary Fig. 8), which can provide useful constraints on their source regions. For the zircons from the Dengfeng Complex and Songshan Group, the majority are characterised by enrichment of heavy REE (HREE), with relatively high $(Lu/Dy)_N$ ratios and Lu values (Fig. 6).

For zircons from the lower Wufoshan Group, some have low $(Lu/Dy)_N$ ratios and Lu values (Fig. 6), reflecting high-pressure metamorphism associated with garnet (metamorphic garnet signature, MGS) in their source regions. In addition, most analysed zircon rims with metamorphic ages clustering at 2.1–1.85 Ga have enriched HREE, which may be attributed to metamorphism under high-T granulite or amphibolite facies (Supplementary Fig. 8). Therefore, the style of Paleoproterozoic metamorphism recorded by detrital zircons is distinct from that of the Neoarchean. This result matches 1) the global

detrital zircon dataset, which shows a similar age peak of MGS zircon in the Paleoproterozoic[55] (Fig. 6); and 2) more abundant high-pressure metamorphic crustal rocks in this time period[56,57] (Fig. 1a).

2.2. Rutile

Rutile grains from the metaconglomerate (20SS09) and quartzites (20SS01, 20SS05) of the lower Songshan Group are mostly euhedral or subhedral, and are generally aligned to the foliations, with lengths of ~40–130 µm and length/width ratios from 1:1 to 2:1 (Supplementary Fig. 6). Rutile records yield U–Pb ages of ca. 2.50 Ga (n=3), 2.31–2.05 Ga (n=11) and 2.0–1.75 Ga (n=62), with two peaks at 1.95 Ga and 1.87 Ga (Fig. 5e). This peak age of rutile is significantly younger than that of detrital zircon from the same rocks, together with the euhedral to irregular rutile shape and alignment with the foliation, indicating that the majority of young rutile populations (e.g., younger than the upper age limit of deposition—2.30 Ga—of the lower Songshan Group) are of metamorphic origin. This interpretation is supported by other lines of evidence. For instance, pressure-independent Zr-in-rutile geothermometry[58] yields temperatures (469–586 °C, mean 505 °C, n=44) for <2.0 Ga rutiles (Fig. 7, Supplementary Table S2), consistent with estimate of the metamorphic temperature of the lower Songshan Group (see Supplementary Note 4 below). Minor older detrital rutiles (ca. 2.50 Ga) and the metamorphic temperature lower than rutile Pb closure temperature (~600 °C) support that the rutile U–Pb isotope has not been reset.

Rutile from quartz sandstone sample 20DF03 of the Ma'anshan Formation of the lower Wufoshan Group are mostly rounded, with lengths of ~60–150 µm and length/width ratios from 1:1 to 2:1 (Supplementary Fig. 6). The rutile morphology, along with the unmetamorphosed host quartz

sandstone, indicates their detrital origin, thus recording metamorphic information of their source regions. The analyses are dominated by Paleoproterozoic ages, with the main populations (n=54) clustering at 1.94 Ga and 1.83 Ga, and minor peaks at 2.32 Ga and 2.15 Ga. The YSG, YC1σ and YC2σ ages are 1746 ± 28 Ma, 1770 ± 10 Ma (n=10) and 1802 ± 6 Ma (n=23), respectively. Relative to rutile from the Songshan Group, rutile from the Wufoshan Group yields a much higher average Zr-in-rutile temperature for <2.0 Ga rutile (~577–876 °C, mean 747 °C, except for one 479 °C, Fig. 7, Supplementary Table S2).

3. $^{40}Ar/^{39}Ar$ geochronology

3.1. Analytical method

The $^{40}Ar/^{39}Ar$ analyses were conducted at the Western Australian Argon Isotope Facility at Curtin University, Australia. The selected amphibole and mica grains were leached in diluted (5N) HF for one minute and then rinsed using distilled water in an ultrasonic bath. After rinsing, minerals were loaded into aluminium discs that were Cd-shielded to minimize undesirable nuclear interference reactions, and irradiated for 40 h in the OSU TRIGA nuclear reactor (Oregon State University, USA), in a central position. The J-values calculated from the GA1550 standard grains within the surrounding pits yielded a value of 0.01096.

The amphibole and mica grains were step-heated using a continuous 100 W PhotonMachine© CO_2 (IR, 10.4 μm) laser fired on the crystals for 33 seconds. Each standard crystal was fused in a single step. The gas was purified in an extra low-volume stainless steel extraction line of 240cc, using two SAES AP10 and one GP50 getter. Ar isotopes were measured in static mode using a low volume (600

cc) ARGUS VI mass spectrometer from Thermofisher© set with a permanent resolution of ~200[59]. We measured the relative abundance of each mass simultaneously using 10 cycles of peak-hopping and 33 s of integration time for each mass. The raw data were processed using the ArArCALC software[60]. The relative abundance of the Ar isotopic data is provided in Supplementary Data 3 and has been corrected for blanks, and mass discrimination.

3.2. $^{40}Ar/^{39}Ar$ age

Rocks from the Dengfeng Complex recorded Neoarchean metamorphism during arc–continent collision as revealed by zircon and titanite U–Pb dating on amphibolite facies rocks[18,42]. The assembled litho-tectonic units of the Dengfeng Complex, together with the overlying Songshan Group, may have also undergone Paleoproterozoic tectono-thermal overpinting as recorded by minor ca. 1.9 Ga metamorphic zircon (n = 2)[61]. We extracted amphibole and mica grains from two amphibolites (DF10-1, DF20-1-5) and from two garnet quartz-mica schists (17DF20-1a, 17DF20-1b) from the Dengfeng Complex for $^{40}Ar/^{39}Ar$ dating, in order to test if they record lower-grade metamorphic overprinting. The metamorphic *P*–*T* estimates are referred to ref[42], which are consistent with high greenschist to amphibolite facies metamorphism with garnet-bearing rocks preserving two generations of zoning[42,61].

The amphibole grains from the amphibolite samples DF10-1 and DF20-1-5 yield $^{40}Ar/^{39}Ar$ plateau ages of 2035 ± 6 Ma (MSWD = 1.1, p = 0.33) and 1861 ± 4 Ma (MSWD = 1.65, p = 0.06), respectively (Supplementary Fig. 9a, b). The white mica grains from the garnet mica-quartz schist samples 17DF20-1a and 17DF20-1b yield two $^{40}Ar/^{39}Ar$ plateau ages of 1826 ± 2 Ma (MSWD = 0.45, p = 0.92) and 1816 ± 3 Ma (MSWD = 1.7, p = 0.16), respectively (Supplementary Fig. 9c, d).

4. Estimation of metamorphic *P–T* conditions

4.1. Single mineral geothermometer

The Songshan Group mainly consists of thick quartzites and quartz mica schists, with greenschist and locally lower amphibolite facies metamorphic grades. The mica quartz schists in the Songshan Group are foliated, with a mineral assemblage dominated by quartz, mica (mainly muscovite with minor biotite), feldspar (mainly K-feldspar and albite), and minor zircon, magnetite, rutile, and apatite (Supplementary Fig. 3f–g). Due to the lack of garnet in the Songshan Group, little appropriate mineral geothermobarometer can be used to estimate the *P–T* conditions, except for single mineral geothermometers like Ti-in-biotite/muscovite geothermometers. The Ti-in-biotite (TiB) geothermometer[62] is calibrated for TiO_2-saturated, rutile- and/or ilmenite-bearing metapelites under wide *P–T* (450–840 °C/1–19 kbar) and compositional ranges, with a random error of ± 65 °C. The mineral chemical composition was analysed using electron microprobe JEOL JXA8230 at the School of Earth Sciences, China University of Geosciences, Wuhan, following the same procedure provided by ref[63]. The analytical error is ± 2 wt.%.

Biotite occurs in quartz mica schists (e.g., 20SS02, 21SS36) from the Wuzhiling Formation of the lower Songshan Group. The samples 20SS02 and 21SS36 have similar mineral assemblages, consisting mainly of quartz, muscovite, and K-feldspar, with minor biotite, monazite, and rutile (Supplementary Fig. 3f–g). When assuming *P* at ~4–6 kbar (based on approximate estimate of *P* of garnet quartz mica schists from the underlying Dengfeng Complex[61]), the temperatures calculated by TiB geothermometer are 578–627 °C (average ~598–616 °C, n=20) and 541–592 °C (average ~558–

574 °C, n=25) for quartz mica schist samples 20SS02 and 21SS36, respectively (Supplementary Table S3). The TiB temperature ranges overlap the Zr-in-rutile temperatures (469–586 °C, average = 505 °C) of < 2.0-Ga metamorphic rutile from the Songshan Group, although the averages are slightly higher (Supplementary Table S2).

Another temperature estimate is obtained by Ti-in-muscovite (TiM) geothermometer[64], which is calibrated for ilmenite- and Al_2SiO_5-bearing metapelites (muscovite Ti = 0.01–0.07, Mg# = 0.05–0.73) at 450–800 °C and 0.1–1.4 GPa, with an error of ± 65 °C. Due to a lack of observation of ilmenite and Al_2SiO_5 minerals, this estimate can just provide an approximate reference and comparison with the TiB geothermometer. We calculated several representative samples, including quartz mica schist 20SS10 from the Dengfeng Complex below the unconformity, 20SS09 from the metaconglomerate matrix of the lowest Songshan Group, quartzites (20SS01, 20SS05), and several quartz mica schists (20SS02, 20SS06, and 20SS36) (Supplementary Table S3). Assuming *P* at 4–6 kbar, the quartz mica schist 20SS10 from the Dengfeng Complex yields TiM temperatures of 553–589 °C (average ~562–569 °C). Quartz mica schist samples from the lower Sonsghan Group yield two main groups of mean TiM temperatures, including one lower (Group 1, ~526–532 °C for 20SS02, ~586–594 °C for 20SS06, ~561–569 °C for 21SS36) and one higher (Group 2, ~683–691 °C for 20SS02, ~702–711 °C for 20SS06). The quartzite-rich samples including the quartzite (20SS01) and metaconglomerate matrix (Supplementary Fig. S3b, dominantly quartz + mica, 20SS09) from the lower Songshan Group contain low Ti muscovite in that is beyond the calibration range of Ti-in-muscovite geothermometer, and they yield lower temperatures of 316–416 °C (mean = ~360 °C, n=9) and 194–340 °C (mean = ~270 °C, n=23), respectively, which are notably different from the mean temperature of 710–719 °C (n=6) of

another quartzite sample 20SS05. The different muscovite compositions and thus temperatures may reflect the different bulk compositions and possible multiple origins of muscovite (e.g., detrital origin, peak and/or retrograde metamorphic origins). The 'high-*T*' muscovite (e.g. >600 °C) is generally associated with K-feldspar, which is likely of detrital origin (e.g., derived from K-rich granite) and was preserved and oriented during greenschist to locally amphibolite facies metamorphism.

The mean temperature (526–586 °C, assuming 4 kbar) of quartz mica schists obtained by the TiM geothermometer from Group 1 muscovite is in agreement with the temperature (average 558–598 °C at 4 kbar) obtained by the Ti-in-biotite geothermometer (Supplementary Table S3). In addition, the Zr-in-rutile geothermometer yields similar temperature ranges (469–586 °C, average 505 °C) for <2.0 Ga metamorphic rutile from quartzites (Supplementary Table S2), implying that the estimation of the metamorphic temperature of the Songshan Group to be ~470–600 °C is reasonable. The peak metamorphic temperature (<600 °C) is lower than the closure temperature of the rutile Pb isotopic system[65], indicating rutile might have continuously grown and been preserved during prolonged orogenesis (from thickening to cooling) from ca. 2.0 Ga to 1.8 Ga.

4.2. Phase equilibria modelling

In order to investigate the *P*–*T* range of the observed mineral assemblage, we further conducted phase equilibria modelling using GeoPS[66], with the internally consistent thermodynamic datasets of ds62[67], a chemical system of MnNCKFMASHTO, and the mineral activity–composition (*a*–*x*) model following ref[68]. The bulk composition is based on XRF analysis. The H_2O is based on LOI, and $Fe^{3+}/\sum Fe^{2+}$ is set to 0.1. The detailed procedure followed ref[42].

The phase diagram of a representative quartz mica schist sample (20SS06) shows that rutile is stable over a large *P*–*T* range below the solidus (Supplementary Fig. S11), suggesting that rutile can possibly form and/or preserve during greenschist to amphibolite facies conditions in such rocks. The characteristic mineral assemblage (quartz–muscovite–K-feldspar–albite–rutile, Supplementary Fig. S3g) is stable under a large field of <620 °C/<12 kbar as constrained by the absence of garnet, plagioclase and melt (Supplementary Fig. S11). The precise constraint on *P*–*T* conditions (in particular *P*) is difficult due to the lack of garnet and low confidence about the intersection of other mineral composition isopleths. For instance, the Si of white mica in sample 20SS06 ranges from 3.32 to 3.44 c.p.f.u. (based on 11 oxygen atoms), which would yield relatively high pressure up to 7.6–9.2 kbar at a rutile ZIR temperature of ~505 °C. If considering the absence of plagioclase and garnet in this sample, then a relatively wide metamorphic pressure range of ~3.6–9.2 kbar can be fairly inferred based on the intersection between the plagioclase- and garnet-in lines and the Zr-in-rutile temperature.

Collectively, we infer that our samples from the lower Songshan Group may have undergone greenschist to lower amphibolite facies metamorphism, with a wide *P*–*T* condition of 470–600 °C/~3.6–9.2 kbar. Considering the different burial levels of the crust during orogenesis, lower or higher *P* and/or *T* conditions may be expected for other rocks of the Songshan Group within lower- or higher-strain zones.

Supplementary Figures

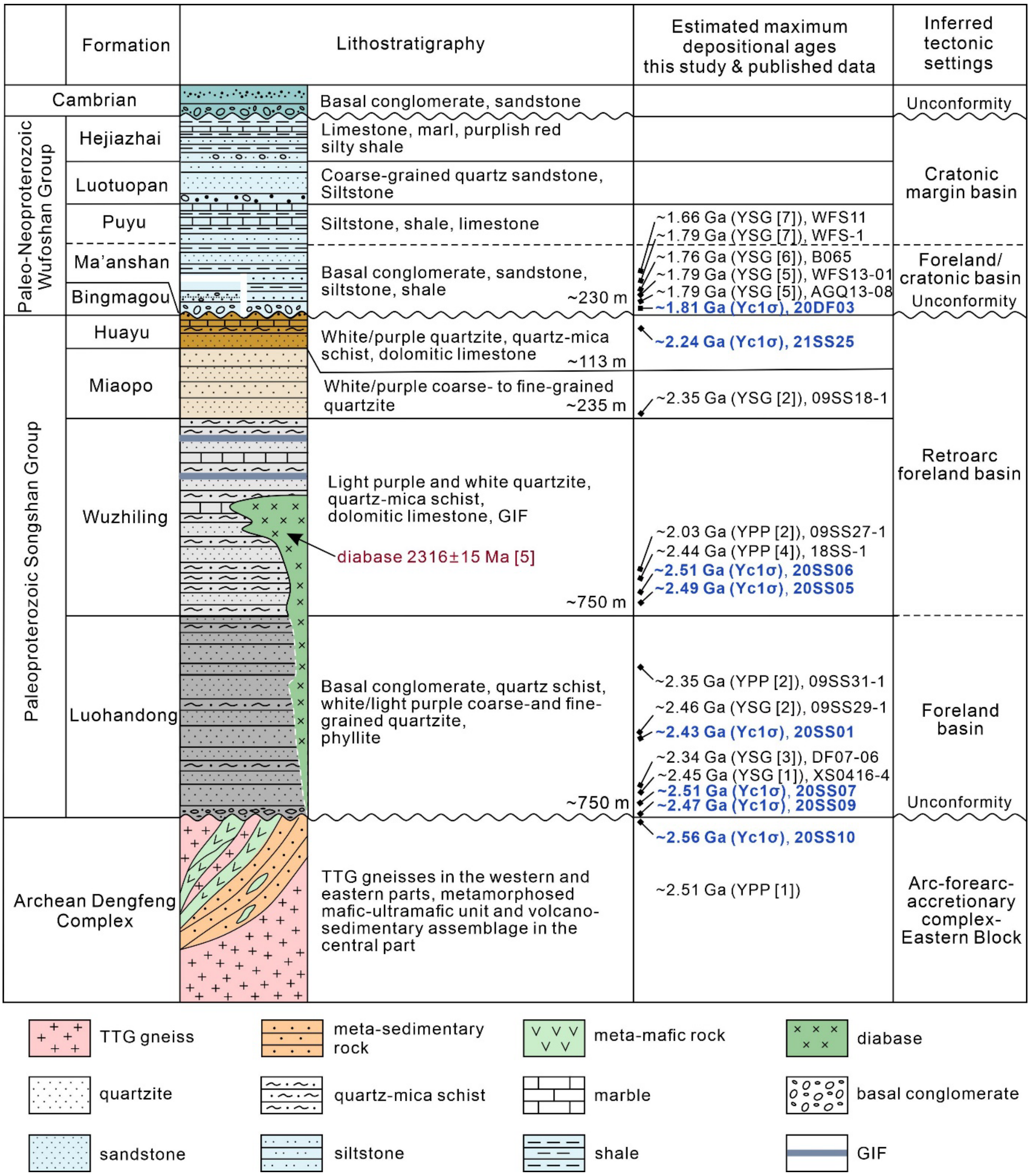

Supplementary Figure 1. Stratigraphic column and inferred MDA (maximum depositional age) of the Songshan and Wufoshan groups in the North China Craton (revised from ref.[27]). Method: YC1σ––Youngest cluster overlapping in age with 1σ. YPP—Youngest probability peak. YSG—Youngest single grain. Blue samples are from this study. Data source: [1]—Wan et al.[51]; [2]—Liu et al.[49]; [3]––Diwu et al.[50]; [4]—Lan et al.[69]; [5] Zhang et al.[70]; [6] Meng et al.[71]; [7] Hu et al.[72]. Note that zircon analyses with discordance > 5% in literature were precluded in the recalculation of MDA.

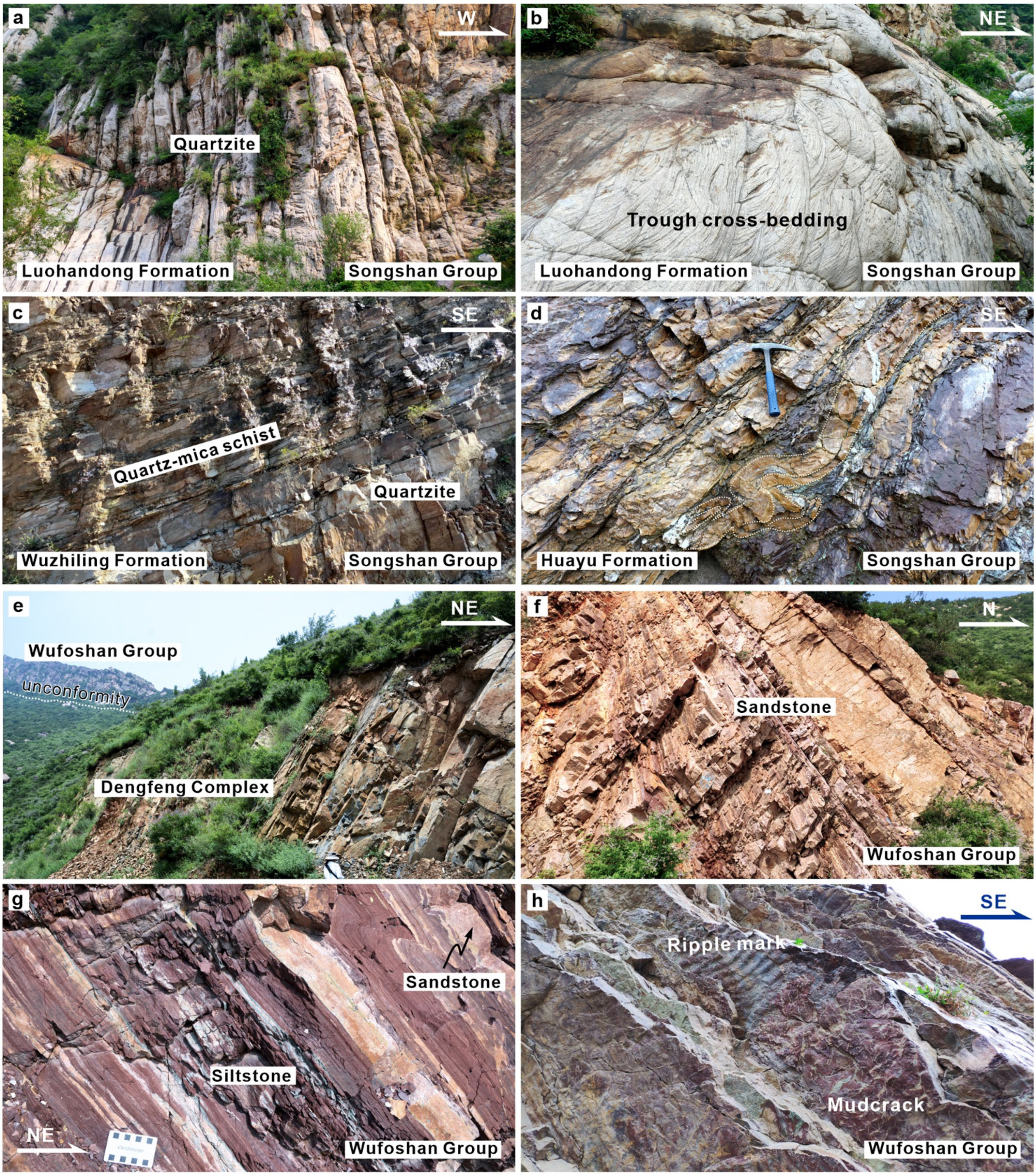

Supplementary Figure 2. Field photographs showing the main lithologies of the Songshan (**a–d**) and Wufoshan (**e–h**) groups. **a** Thick white quartzite in the Luohandong Formation of the lower Songshan Group. **b** Trough cross-bedding within the Luohandong Formation. **c** Quartz mica schist and quartzite in the Wuzhiling Formation of the lower Songshan Group. **d** Purple to white quartzite and quartz mica schist in the Huayu Formation of the upper Songshan Group. **e** Unconformity between the Wufoshan Group and the Neoarchean Dengfeng Complex. **f** Quartz sandstone in the

Ma’anshan Formation of the lower Wufoshan Group (sample 20DF03). **g** Purple to white sandstone–siltstone in the Ma’anshan Formation. **h** Ripple marks and mudcrack in the Ma’anshan Formation.

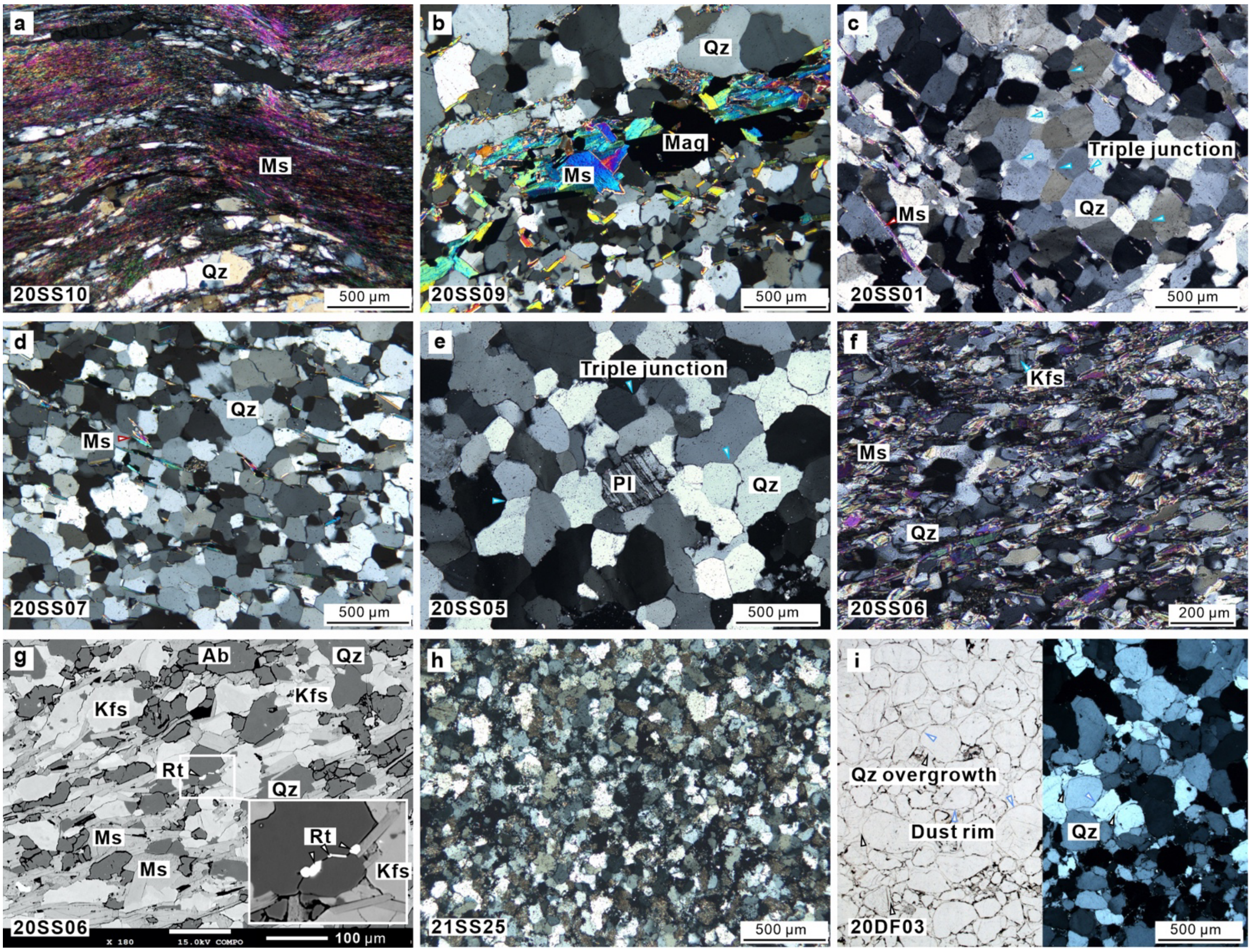

Supplementary Figure 3. Photomicrographs showing zircon U–Pb dated samples, including the quartz-mica schist of the Dengfeng Complex (**a**), metaconglomerate (**b**), quartzites (**c**–**e**, **h**) and quartz-mica schist (**f**–**g**) of the Songshan Group, and quartz sandstone (**i**) of the lower Wufoshan Group. Note that the quartz texture of the quartz sandstone of the lower Wufoshan Group is distinct from the recrystallised quartz from the quartzites of the Songshan Group. Mineral abbrevations: Ab–albite, Ms–muscovite, Pl–plagiocalse, Kfs–K-feldspar, Rt–rutile, Qz–quartz, Mag–magnetite.

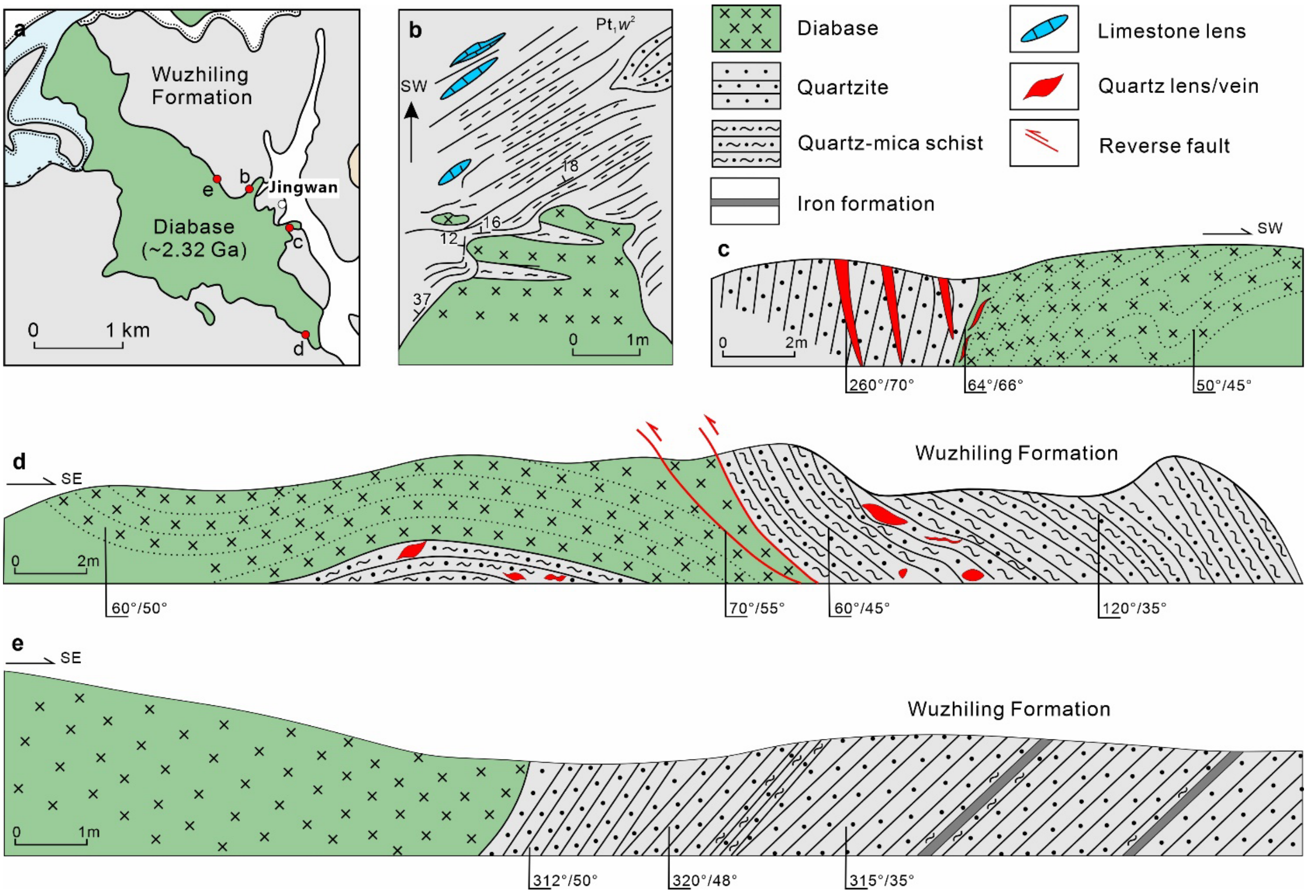

Supplementary Figure 4. Litho-structural maps showing the intrusion relation between ca. 2.32-Ga diabase plutons[30] and the Wuzhiling Formation of the Songshan Group. The red dots in (**a**) indicate the locations shown in (**b**)–(**e**).

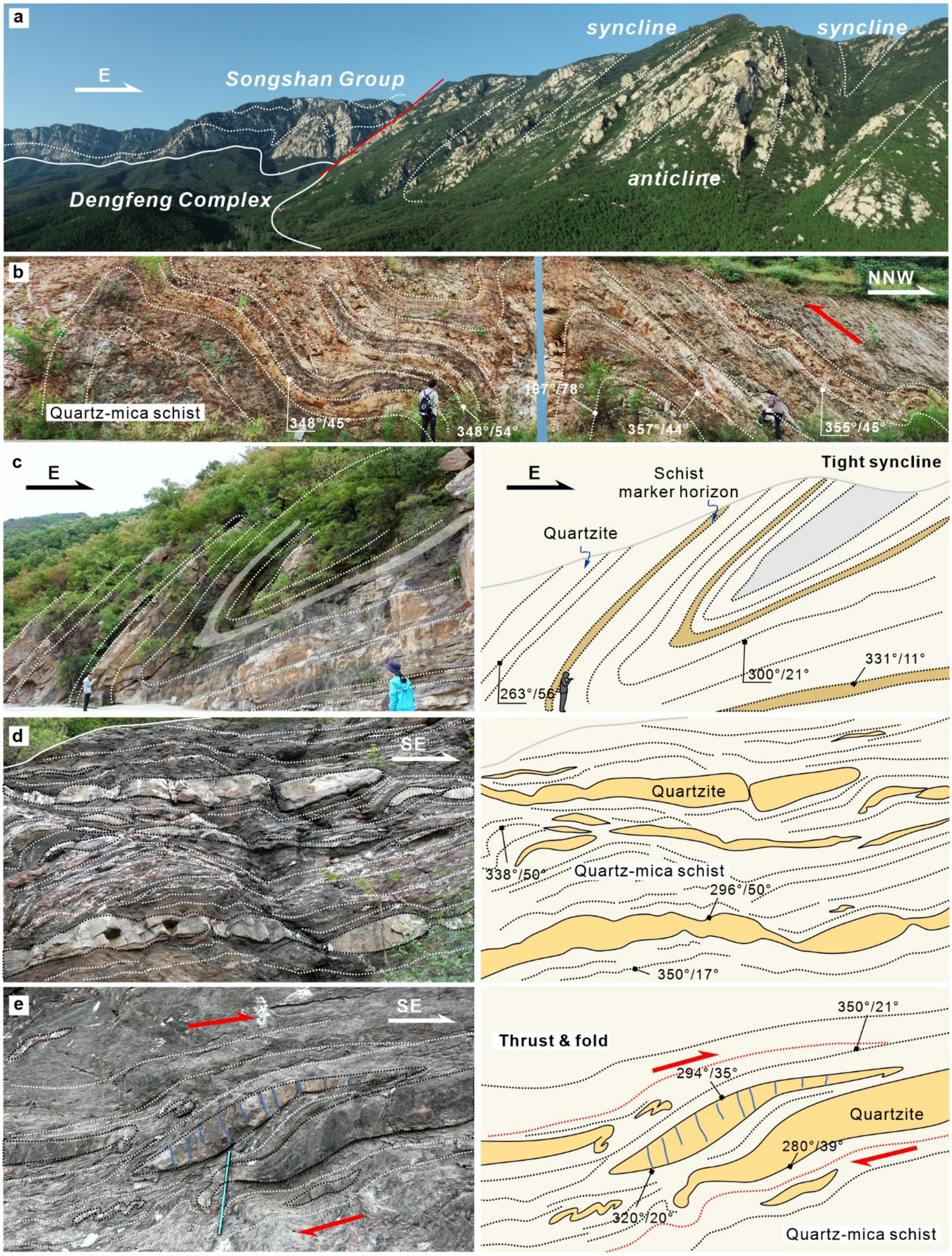

Supplementary Figure 5. Field photographs and sketches showing regional and outcrop-scale fold and thrust structures in the Songshan Group. **a** Regional scale composite folds of the lower Songshan Group, and unconformity between the Songshan Group and the underlying Archean Dengfeng

Complex. **b** Folds within the Wuzhiling Formation. **c** Large isoclinal overturned tight folds within the Wuzhiling Formation. **d** Boudinaged and fragmented quartzite layers within highly-deformed quartz-mica schist in the Wuzhiling Formation. **e** Top-to-the-SE sense of thrusting marked by the asymmetric quartzite boudinage and small-scale folds.

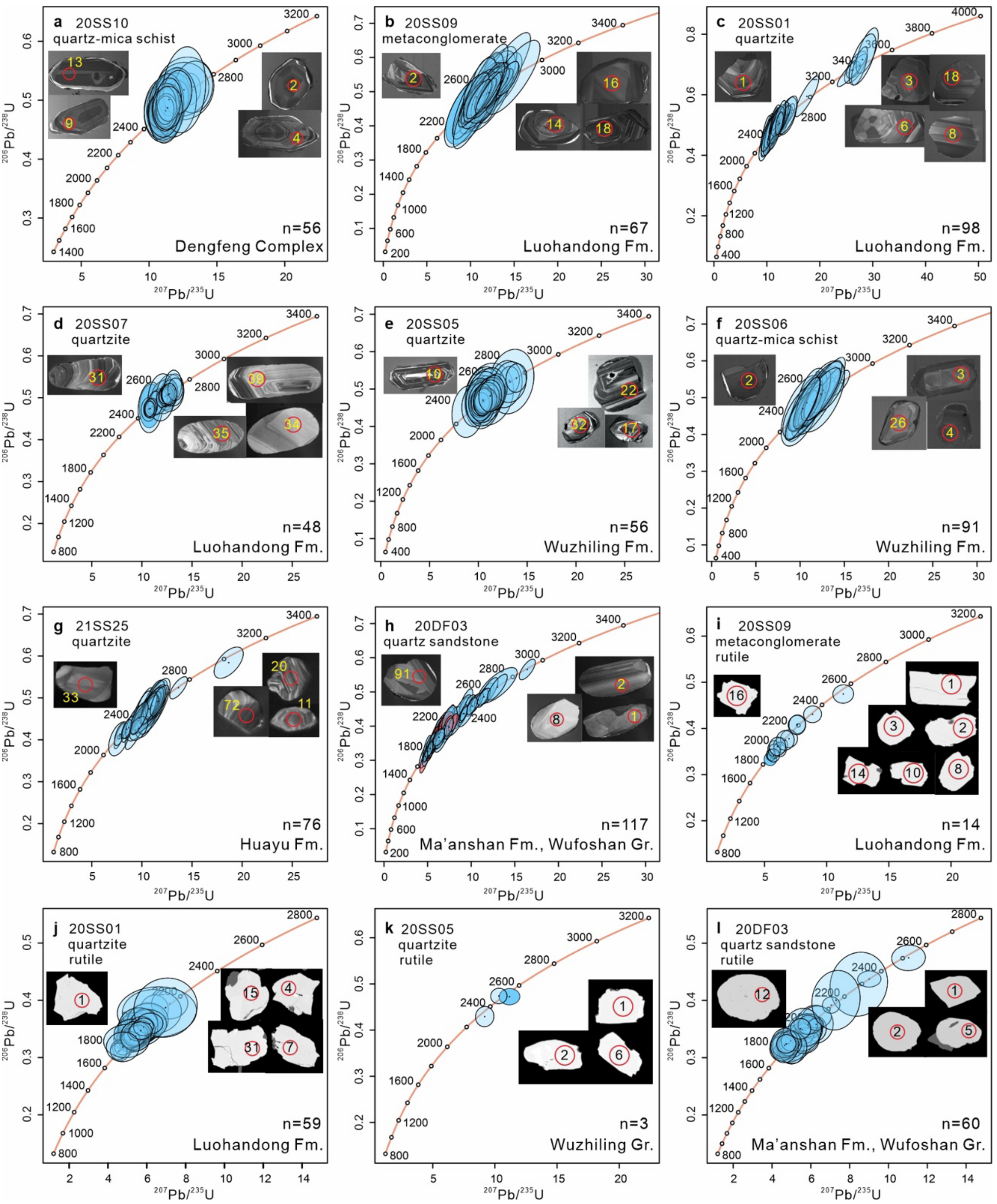

Supplementary Figure 6. Concordia diagrams of U–Pb dates and characteristic zircon and rutile grains in CL or BSE images. **a** quartz mica schist sample 20SS10 from the Dengfeng Complex. **b, i** basal metaconglomerate sample 20SS09 from the lowest Songshan Group. **c, j** quartzite sample 20SS01 from the Luohandong Formation of the lower Songshan Group. **d** quartzite sample 20SS07

from the Luohandong Formation of the lower Songshan Group. **e, k** quartzite sample 20SS05 from the Wuzhiling Formation of the lower Songshan Group. **f** quartz mica schist sample 20SS06 from the Wuzhiling Formation of the Songshan Group. **g** quartzite sample 21SS25 from the Huayu Formation of the upper Songshan Group. **h, l** quartz sandstone sample 20DF03 from the Ma'anshan Formation of the Wufoshan Group.

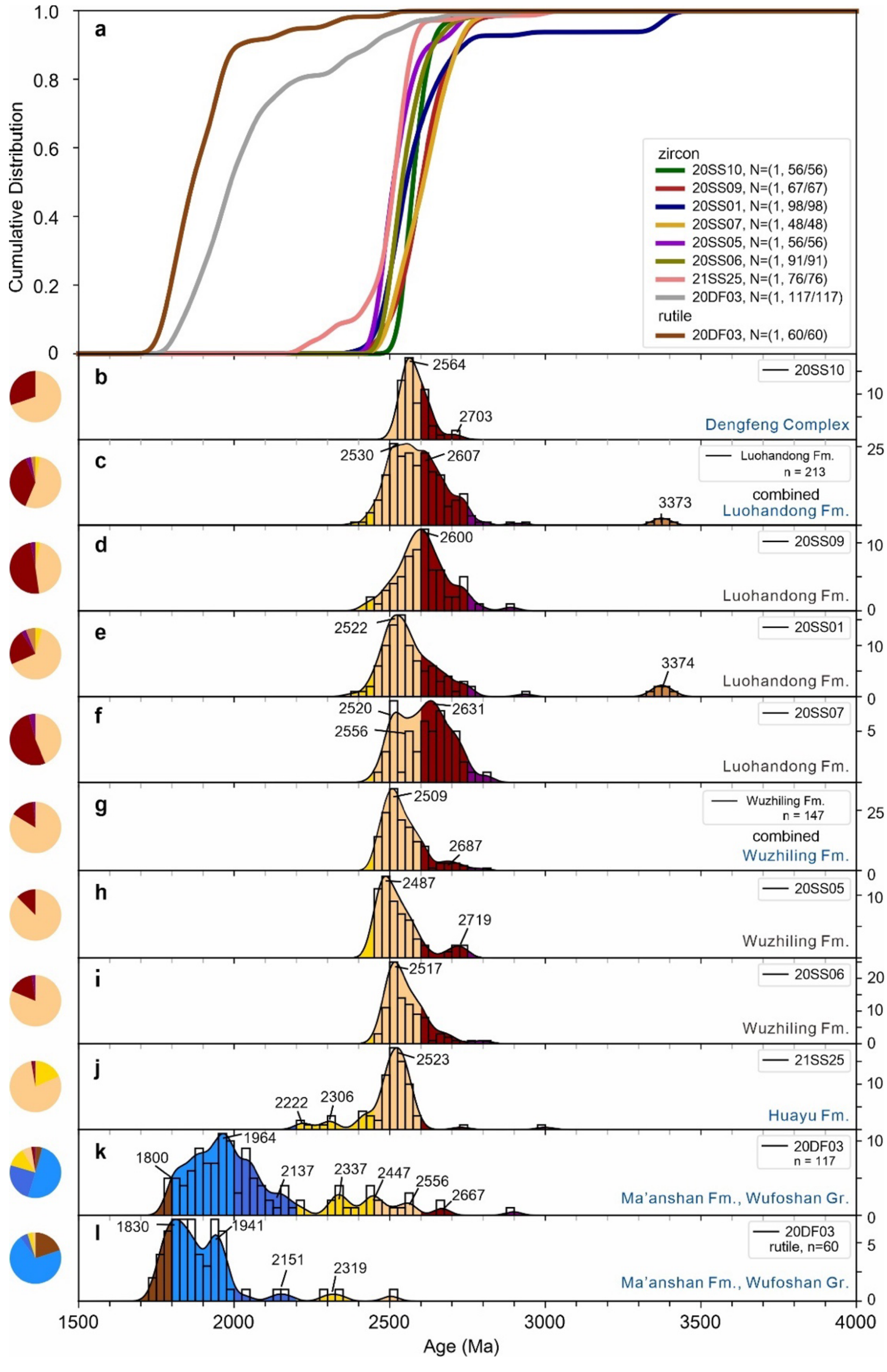

Supplementary Figure 7. Cumulative distribution (**a**) and Kernel density estimates (KDE, **b–l**) of ages of zircon and rutile for the individual sample. **a** Cumulative distribution curves of zircon and rutile ages. **b** Zircon age distribution of the sample 20SS10 from the Dengfeng Complex. **c–f** Zircon age distribution of the Luohandong Formation (Fm) of the Songshan Group. **g–i** Zircon age distribution of the Wuzhiling Formation of the Songshan Group. **j** Zircon age distribution of the

Huayu Formation of the Songshan Group. **k** Zircon age distribution of the Ma'anshan Formation of the Wufoshan Group. **l** Rutile age distribution of the Ma'anshan Formation of the Wufoshan Group.

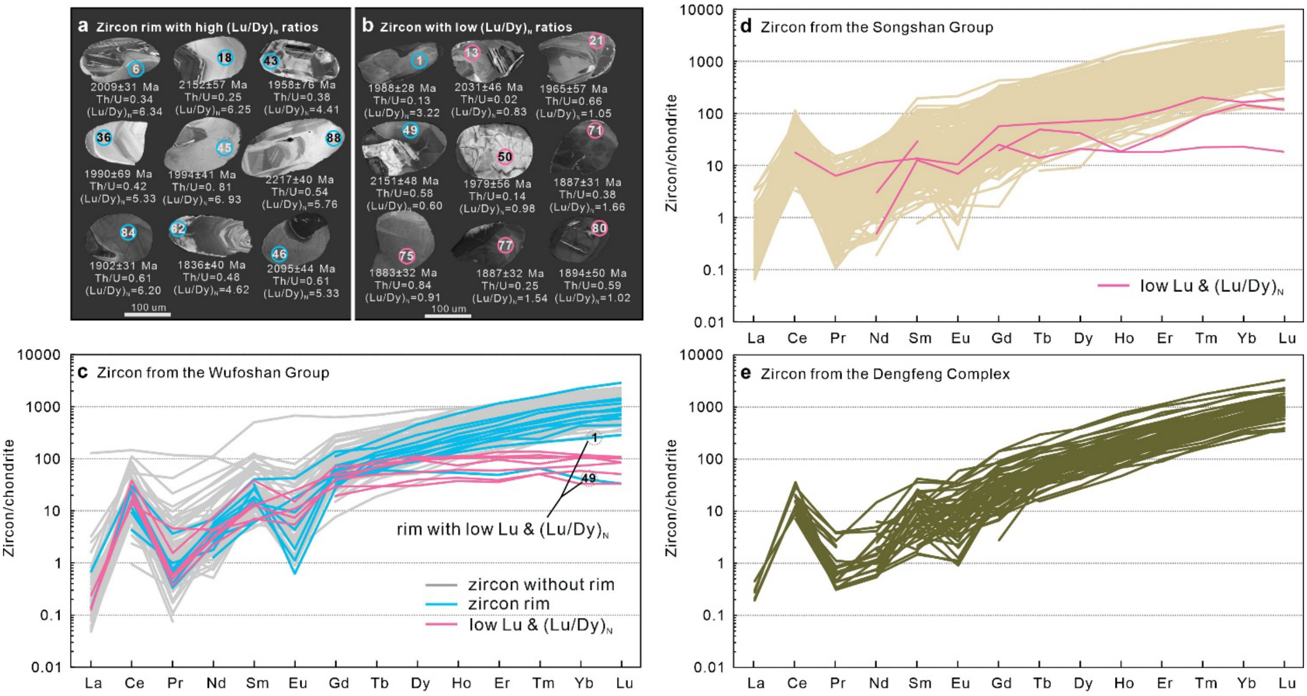

Supplementary Figure 8. (**a–b**) Representative CL images of metamorphic zircons and (**c**) chondrite-normalised rare earth element (REE) patterns of zircons from the Wufoshan Group; (**d**) REE patterns of zircons from the Songshan Group; (**e**) REE patterns of zircons from the Dengfeng Complex. The near-flat-HREE zircon [low Lu and $(Lu/Dy)_N$] is interpreted to have formed during the growth of garnet during relatively high-pressure (>1 GPa) metamorphism[55]. Note that the majority of zircon rims analysed have enriched HREE, which is common in zircon under high-T granulite or amphibolite facies metamorphism.

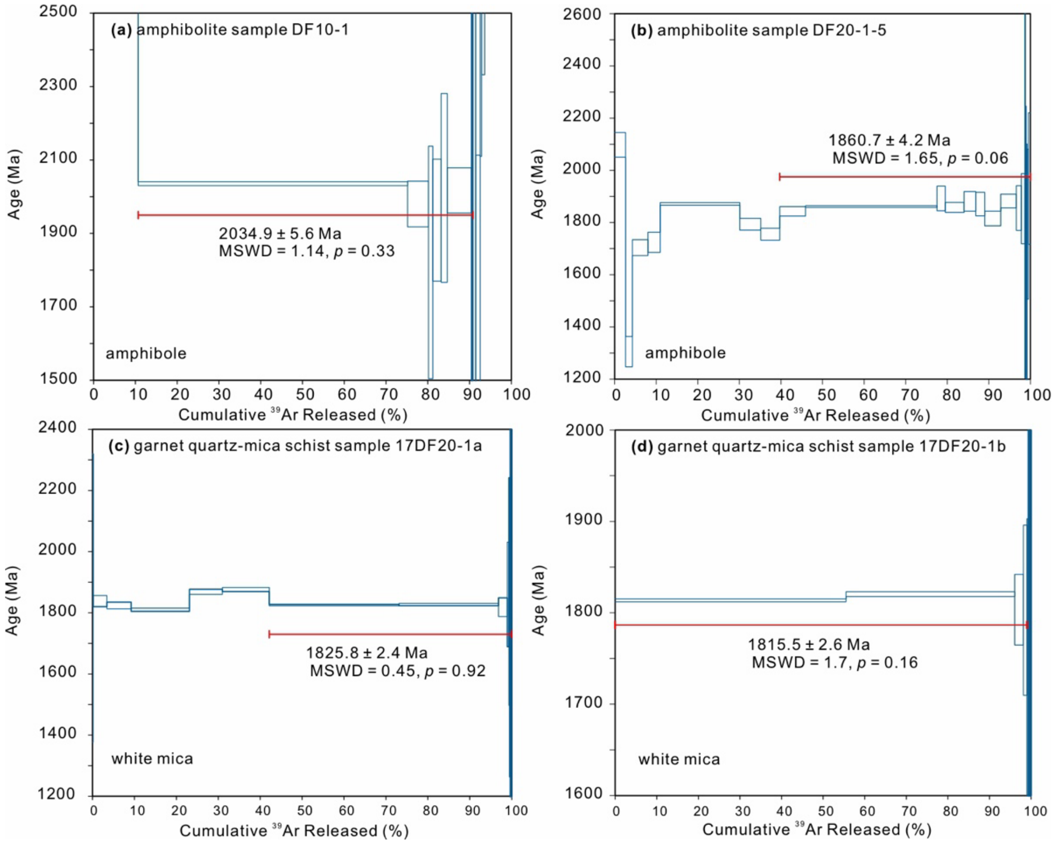

Supplementary Figure 9. $^{40}Ar/^{39}Ar$ plateau ages of amphibole and white mica from the Dengfeng Complex. **a** amphibolite sample DF10-1. **b** amphibolite sample DF20-1-5. **c–d** quartz mica schist samples 17DF20-1a and 17DF20-1b.

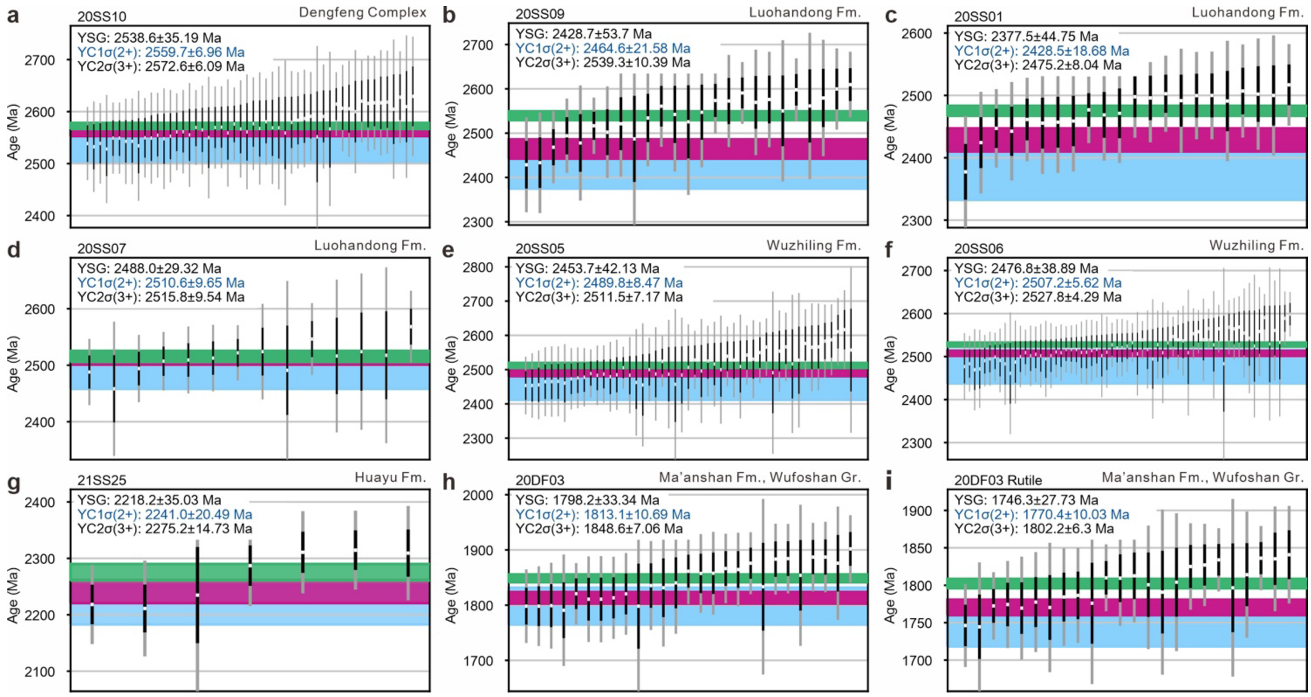

Supplementary Figure 10. Estimates of maximum depositional ages (MDA) for different samples calculated by detrital zircon/rutile U–Pb ages. **a** quartz mica schist sample 20SS10 from the Dengfeng Complex. **b** basal metaconglomerate sample 20SS09 from the lowest Songshan Group. **c** quartzite sample 20SS01 from the Luohandong Formation of the lower Songshan Group. **d** quartzite sample 20SS07 from the Luohandong Formation of the lower Songshan Group. **e** quartzite sample 20SS05 from the Wuzhiling Formation of the lower Songshan Group. **f** quartz mica schist sample 20SS06 from the Wuzhiling Formation of the Songshan Group. **g** quartzite sample 21SS25 from the Huayu Formation of the upper Songshan Group. **h, i** quartz sandstone sample 20DF03 from the Ma'anshan Formation of the Wufoshan Group (**h**, zircon age; **i**, rutile age). YSG, youngest single grain; YC1σ, youngest cluster overlapping in age at 1σ; YC2σ, youngest cluster overlapping in age at 2σ. The MDA methods followed refs[36, 69]. The calculated uncertainties are at 1σ.

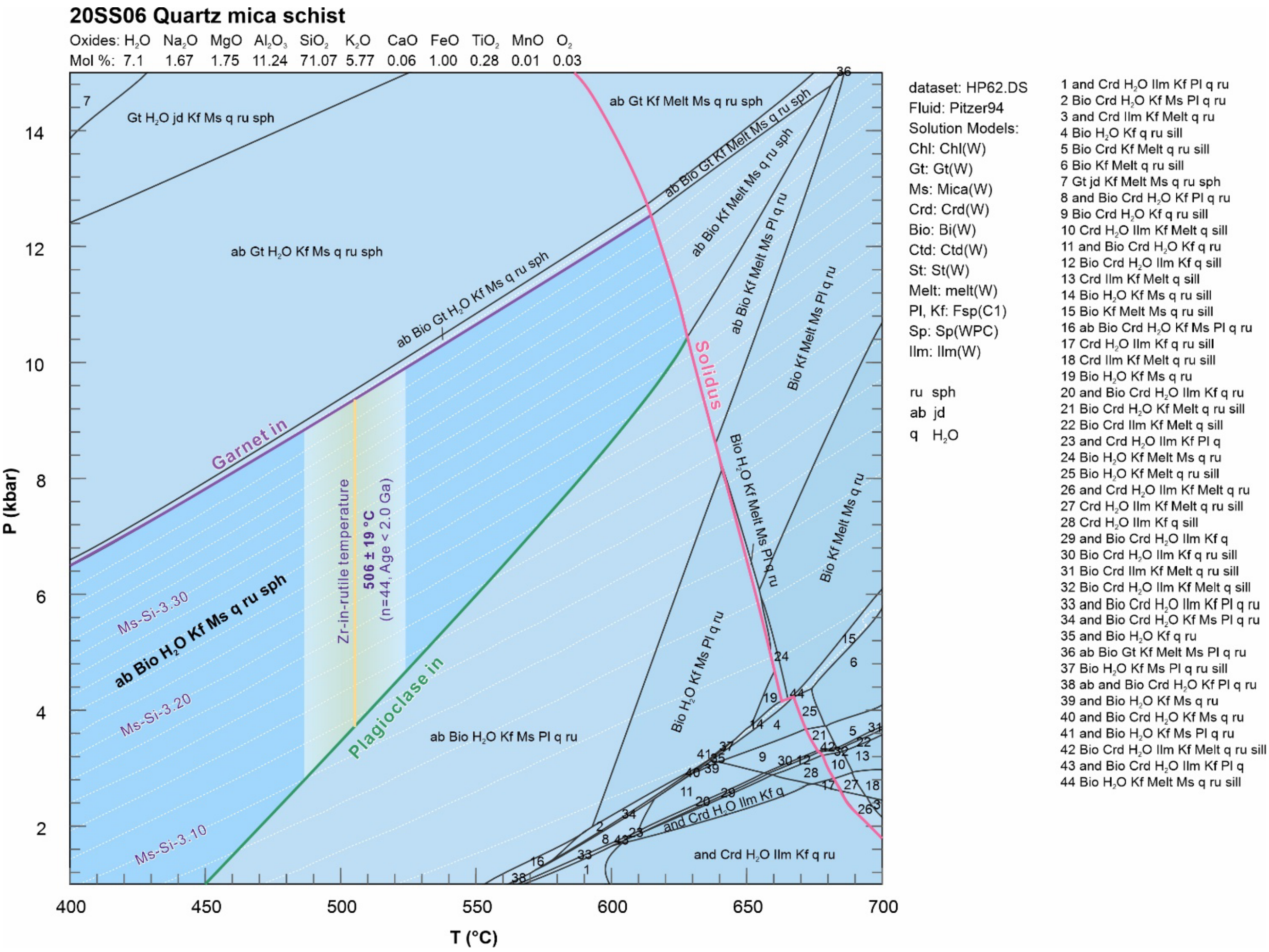

Supplementary Figure 11. Phase equilibria modelling using bulk compositions of a quartz mica schist sample 20SS06. The average Zr-in-rutile temperature for metamorphic rutiles (<2.0 Ga) is plotted.

Supplementary Tables

Supplementary Table 1. Maximum depositional age (MDA) of (meta)sedimentary rocks.

Sample	Lithology	Unit	Mineral	Number	YSG		YC1σ				YC2σ			
					Age	1σ error	WMA	1σ error	MSWD	cluster size	WMA	1σ error	MSWD	cluster size
20SS10	QMS	Dengfeng Complex	zircon	56	2539	35	**2560**	**7**	0.13	39	2573	6	0.38	52
20SS09	Metaconglomerate matrix	LHD, SG	zircon	67	2429	54	**2465**	**22**	0.32	5	2539	10	1.37	19
20SS01	Quartzite	LHD, SG	zircon	98	2378	45	**2429**	**19**	0.6	4	2475	8	0.76	19
20SS07	Quartzite	LHD, SG	zircon	48	2488	29	**2511**	**10**	0.28	13	2516	10	0.7	10
20SS05	Quartzite	WZL, SG	zircon	56	2454	42	**2490**	**8**	0.24	34	2512	7	0.68	51
20SS06	QMS	WZL, SG	zircon	91	2477	39	**2507**	**6**	0.21	35	2528	4	0.85	59
21SS25	Quartzite	HY, SG	zircon	76	2218	35	**2241**	**20**	0.86	4	2275	15	1.36	7
20DF03	Quartz sandstone	Wufoshan Group	zircon	117	1798	33	**1813**	**11**	0.14	13	1849	7	0.98	24
20DF03	Quartz sandstone	Wufoshan Group	rutile	60	1746	28	**1770**	**10**	0.18	10	1802	6	0.92	23

Note: YSG—Youngest single grain; YC1σ—Youngest cluster overlapping in age with 1σ uncertainty; YC2σ—Youngest cluster overlapping in age with 2σ uncertainty. QMS—Quartz mica schist. SG—Songshan Group, LHD—Luohandong Formation, WZL—Wuzhiling Formation, HY––Huayu Formation.

Supplementary Table 2. Summary of rutile ages and Zr-in-rutile temperatures.

Sample No.	Lithology	Rutile age (Ga)		Zr-in-rutile (°C)			
		**Metamorphic rutile**	**Detrital rutile**	**Range**	**Average**	**Peak**	**Range and Average of ca. 2.0–1.75 Ga rutile**
Songshan Group							
20SS09	Matrix of metaconglomerate	1.86–2.00, WMA=1.93, n=8; 2.05–2.31, n=5	2.50, n=1	485–586	526, n=10	518	506–586, average 529, n=7
20SS01	Quartzite	1.75–1.88, WMA=1.83, n=21; 1.89–2.0, WMA=1.93, n=33; 2.07–2.14, WMA=2.10, n=5	-	469–528	500, n=37	503	469–528, average 500, n=37
20SS05	Quartzite	2.30, n=1	2.49–2.50, n=2	656–765	693, n=3	-	-
All rutile from the Songshan Group		1.75–1.78,WMA=1.76, n=5; 1.82–1.90, WMA=1.87, n=27; 1.91–2.0, WMA=1.95, n=30; 2.05–2.14, WMA=2.08, n=7; 2.21–2.31, n=4	2.49–2.50, n=3	469–765	**517, n=50**	503	**469–586, average 505, n=44**
Wufoshan Group							
20DF03	Quartz sandstone	-	1.75–1.90, WMA = 1.83, n=37 1.92–1.98, WMA=1.94, n=17 2.04, 2.13, 2.17, 2.30, 2.34, 2.50, n=6	479–876	**734, n=40**	754	**479–876, average 740, n=36**

Note: WMA—weighted mean age. Due to the uncertain pressure of detrital and metamorphic rutiles, the Zr-in-rutile geothermometer follows pressure-independent calibration by Ferry and Waston[58], which yields temperatures comparable with those by pressure-dependent calibration (α-quartz) by Kohn[73] in the most temperature range when considering a pressure 13 ± 5 kbar with error.

Supplementary Table 3. Summary of metamorphic temperature estimated by Ti-in-mica geothermometer.

Sample No.	Lithology	Unit	Ti-in-biotite (°C)				Ti-in-muscovite (°C)			
			Range		Average		Range		Average	
			4 kbar	6kbar	4 kbar	6kbar	4 kbar	6kbar	4 kbar	6kbar
Dengfeng Complex										
20SS10	QMS	Dengfeng Complex	-	-	-	-	553–582	560–589	562, n=6	569
Songshan Group										
20SS09	Matrix of metaconglomerate	Luohandong Formation	-	-	-	-	194–335	196–340	267, n=23	271
20SS01	Quartzite	Luohandong Formation	-	-	-	-	316–410	320–416	358, n=9	363
20SS05	Quartzite	Wuzhiling Formation	-	-	-	-	678–743	687–753	710, n=6	719
20SS02	QMS	Wuzhiling Formation	578–609	595–627	598, n=20	616	Group 1: 500–555; Group 2: 644–714	506–562; 652–723	526, n=3; 683, n=28	532; 691
20SS06	QMS	Wuzhiling Formation	-	-	-	-	Group 1: 586–586; Group 2: 660–731	594–594; 668–740	586, n=2; 702 , n=20	594; 711
21SS36	QMS	Wuzhiling Formation	541–575	558–592	558, n=25	574	525–606	532–614	561, n=29	569

Note: QMS—quartz mica schist. The temperature by Ti-in-biotite (TiB) geothermometer[62] is preferred, while the temperature by Ti-in-muscovite geothermometer[64] is used for reference and comparison.

Supplementary References

1. Kusky, T. M. Geophysical and geological tests of tectonic models of the North China Craton. *Gondwana Res.* **20**, 26-35 (2011).
2. Kusky, T. M. & Li, J. Paleoproterozoic tectonic evolution of the North China Craton. *J. Asian Earth Sci.* **22**, 383-397 (2003).
3. Peng, P. et al. Spatial distribution of ~1950–1800Ma metamorphic events in the North China Craton: Implications for tectonic subdivision of the craton. *Lithos*. **202-203**, 250-266 (2014).
4. Polat, A. et al. Geochemistry of Neoarchean (ca. 2.55–2.50 Ga) volcanic and ophiolitic rocks in the Wutaishan greenstone belt, central orogenic belt, North China craton: Implications for geodynamic setting and continental growth. *Geol. Soc. Am. Bull.* **117**, 1387-1399 (2005).
5. Tang, L. & Santosh, M. Neoarchean-Paleoproterozoic terrane assembly and Wilson cycle in the North China Craton: an overview from the central segment of the Trans-North China Orogen. *Earth-Sci. Rev.* **182**, 1-27 (2018).
6. Zhai, M., Guo, J. & Liu, W. Neoarchean to Paleoproterozoic continental evolution and tectonic history of the North China Craton: a review. *J. Asian Earth Sci.* **24**, 547-561 (2005).
7. Zhai, M. et al. Precambrian key tectonic events and evolution of the North China craton. *Geological Society, London, Special Publications*. **338**, 235-262 (2010).
8. Zhai, M. & Santosh, M. The early Precambrian odyssey of the North China Craton: A synoptic overview. *Gondwana Res.* **20**, 6-25 (2011).
9. Zhao, G. et al. Amalgamation of the North China Craton: Key issues and discussion. *Precambrian Res.* **222-223**, 55-76 (2012).
10. Zhao, G., Sun, M., Wilde, S. A. & Li, S. Late Archean to Paleoproterozoic evolution of the North China Craton: key issues revisited. *Precambrian Res.* **136**, 177-202 (2005).
11. Zhao, G., Wilde, S. A., Cawood, P. A. & Sun, M. Archean blocks and their boundaries in the North China Craton: lithological, geochemical, structural and P–T path constraints and tectonic evolution. *Precambrian Res.* **107**, 45-73 (2001).
12. Chen, H. et al. Metamorphism and geochronology of the Luoning metamorphic terrane, southern terminal of the Palaeoproterozoic Trans-North China Orogen, North China Craton. *Precambrian Res.* **264**, 156-178 (2015).
13. Qian, J. H. & Wei, C. J. P-T-t evolution of garnet amphibolites in the Wutai-Hengshan area, North China Craton: insights from phase equilibria and geochronology. *J. Metamorph. Geol.* **34**, 423-446 (2016).

14. Xiao, L., Liu, F. & Chen, Y. Metamorphic P–T–t paths of the Zanhuang metamorphic complex: Implications for the Paleoproterozoic evolution of the Trans-North China Orogen. *Precambrian Res.* **255**, 216-235 (2014).
15. Zhang, J. et al. High-pressure mafic granulites in the Trans-North China Orogen: Tectonic significance and age. *Gondwana Res.* **9**, 349-362 (2006).
16. Zhao, G. C., Wilde, S. A., Cawood, P. A. & Lu, L. Z. Petrology and P-T path of the Fuping mafic granulites: implications for tectonic evolution of the central zone of the North China craton. *J. Metamorph. Geol.* **18**, 375-391 (2000).
17. Zhao, G., Cawood, P. & Lu, L. Petrology and P–T history of the Wutai amphibolites: implications for tectonic evolution of the Wutai Complex, China. *Precambrian Res.* **93**, 181-199 (1999).
18. Deng, H. et al. A 2.5 Ga fore-arc subduction-accretion complex in the Dengfeng Granite-Greenstone Belt, Southern North China Craton. *Precambrian Res.* **275**, 241-264 (2016).
19. Deng, H. et al. Geochemistry of Neoarchean mafic volcanic rocks and late mafic dikes in the Zanhuang Complex, Central Orogenic Belt, North China Craton: Implications for geodynamic setting. *Lithos*. **175-176**, 193-212 (2013).
20. Diwu, C., Sun, Y., Guo, A., Wang, H. & Liu, X. Crustal growth in the North China Craton at ~2.5Ga: Evidence from in situ zircon U–Pb ages, Hf isotopes and whole-rock geochemistry of the Dengfeng complex. *Gondwana Res.* **20**, 149-170 (2011).
21. Fu, J. et al. Late Neoarchean monzogranitic–syenogranitic gneisses in the Eastern Hebei–Western Liaoning Province, North China Craton: Petrogenesis and implications for tectonic setting. *Precambrian Res.* **303**, 392-413 (2017).
22. Guo, R. et al. Neoarchean subduction: A case study of arc volcanic rocks in Qinglong-Zhuzhangzi area of the Eastern Hebei Province, North China Craton. *Precambrian Res.* **264**, 36-62 (2015).
23. Huang, X., Wilde, S. A. & Zhong, J. Episodic crustal growth in the southern segment of the Trans-North China Orogen across the Archean-Proterozoic boundary. *Precambrian Res.* **233**, 337-357 (2013).
24. Jiang, N. et al. Archean TTGs and sanukitoids from the Jiaobei terrain, North China craton: Insights into crustal growth and mantle metasomatism. *Precambrian Res.* **281**, 656-672 (2016).
25. Polat, A. et al. Geochemical and petrological evidence for a suprasubduction zone origin of Neoarchean (ca. 2.5 Ga) peridotites, central orogenic belt, North China craton. *Geol. Soc. Am. Bull.* **118**, 771-784 (2006).
26. Tang, L. & Santosh, M. Neoarchean granite-greenstone belts and related ore mineralization in the North China Craton: An overview. *Geosci. Front.* **9**, 751-768 (2018).

27. Wang, C., Song, S., Niu, Y., Wei, C. & Su, L. TTG and Potassic Granitoids in the Eastern North China Craton: Making Neoarchean Upper Continental Crust during Micro-continental Collision and Post-collisional Extension. *J. Petrol.* **57**, 1775-1810 (2016).
28. Wang, W. et al. Neoarchean intra-oceanic arc system in the Western Liaoning Province: Implications for Early Precambrian crustal evolution in the Eastern Block of the North China Craton. *Earth-Sci. Rev.* **150**, 329-364 (2015).
29. Wang, W. et al. Crustal reworking in the North China Craton at ~2.5 Ga: evidence from zircon U-Pb age, Hf isotope and whole rock geochemistry of the felsic volcano-sedimentary rocks from the western Shandong Province. *Geol. J.* **48**, 406-428 (2013).
30. Wang, X., Huang, X., Yang, F. & Luo, Z. Late Neoarchean magmatism and tectonic evolution recorded in the Dengfeng Complex in the southern segment of the Trans-North China Orogen. *Precambrian Res.* **302**, 180-197 (2017).
31. Li, J. & Kusky, T. A Late Archean foreland fold and thrust belt in the North China Craton: Implications for early collisional tectonics. *Gondwana Res.* **12**, 47-66 (2007).
32. Li, S. et al. Deformation history of the Hengshan–Wutai–Fuping Complexes: Implications for the evolution of the Trans-North China Orogen. *Gondwana Res.* **18**, 611-631 (2010).
33. Ma, X. Y., Suo, S. T., You, Z. D. & Liu, R. Q. *Tectonic deformation of the Songshan area, Henan Province, China*. (Publishing House of Geology, Beijing, 1981).
34. Wang, J. et al. A late Archean tectonic mélange in the Central Orogenic Belt, North China Craton. *Tectonophysics*. **608**, 929-946 (2013).
35. Wang, J. et al. Structural relationships along a Neoarchean arc-continent collision zone, North China craton. *Geol. Soc. Am. Bull.* **129**, 59-75 (2017).
36. Zhang, J. et al. Structural pattern of the Wutai Complex and its constraints on the tectonic framework of the Trans-North China Orogen. *Precambrian Res.* **222-223**, 212-229 (2012).
37. Trap, P. et al. The Zanhuang Massif, the second and eastern suture zone of the Paleoproterozoic Trans-North China Orogen. *Precambrian Res.* **172**, 80-98 (2009).
38. Trap, P., Faure, M., Lin, W., Le Breton, N. & Monié, P. Paleoproterozoic tectonic evolution of the Trans-North China Orogen: Toward a comprehensive model. *Precambrian Res.* **222-223**, 191-211 (2012).
39. Zhai, M. Cratonization and the Ancient North China Continent: A summary and review. *Science China Earth Sciences*. **54**, 1110-1120 (2011).
40. Zhai, M. Multi-stage crustal growth and cratonization of the North China Craton. *Geosci. Front.* **5**, 457-469 (2014).

41. Zhai, M. & Peng, P. Origin of early continents and beginning of plate tectonics. *Sci. Bull.* **65**, 970-973 (2020).
42. Huang, B. et al. Paired metamorphism in the Neoarchean: A record of accretionary-to-collisional orogenesis in the North China Craton. *Earth Planet. Sci. Lett.* **543**, 116355 (2020).
43. Huang, B. et al. Coexisting divergent and convergent plate boundary assemblages indicate plate tectonics in the Neoarchean. *Nat. Commun.* **13**, 6450 (2022).
44. Zhai, M. et al. Late Neoarchean magmatic – metamorphic event and crustal stabilization in the North China Craton. *Am. J. Sci.* **321**, 206-234 (2021).
45. Kusky, T. M. et al. Insights into the tectonic evolution of the North China Craton through comparative tectonic analysis: A record of outward growth of Precambrian continents. *Earth-Sci. Rev.* **162**, 387-432 (2016).
46. Kusky, T., Li, J. & Santosh, M. The Paleoproterozoic North Hebei Orogen: North China craton's collisional suture with the Columbia supercontinent. *Gondwana Res.* **12**, 4-28 (2007).
47. Santosh, M., Sajeev, K. & Li, J. H. Extreme crustal metamorphism during Columbia supercontinent assembly: Evidence from North China Craton. *Gondwana Res.* **10**, 256-266 (2006).
48. Sun, G. et al. Thermal state and evolving geodynamic regimes of the Meso- to Neoarchean North China Craton. *Nat. Commun.* **12**, 3888 (2021).
49. Liu, C. et al. Detrital zircon U–Pb dating, Hf isotopes and whole-rock geochemistry from the Songshan Group in the Dengfeng Complex: Constraints on the tectonic evolution of the Trans-North China Orogen. *Precambrian Res.* **192-195**, 1-15 (2012).
50. Diwu, C. et al. U-Pb ages and Hf isotopes for detrital zircons from quartzite in the Paleoproterozoic Songshan Group on the southwestern margin of the North China Craton. *Sci. Bull.* **53**, 2828-2839 (2008).
51. Wan, Y. et al. Early precambrian crustal evolution in the Dengfeng area, Henan province (eastern China): constraints from geochemistry and SHRIMP U–Pb zircon dating. *Acta Geologica Sinica*. **83**, 982-999 (2009).
52. Coutts, D. S., Matthews, W. A. & Hubbard, S. M. Assessment of widely used methods to derive depositional ages from detrital zircon populations. *Geosci. Front.* **10**, 1421-1435 (2019).
53. Dickinson, W. R. & Gehrels, G. E. Use of U–Pb ages of detrital zircons to infer maximum depositional ages of strata: A test against a Colorado Plateau Mesozoic database. *Earth Planet. Sci. Lett.* **288**, 115-125 (2009).
54. Sharman, G. R. & Malkowski, M. A. Needles in a haystack: Detrital zircon U-Pb ages and the maximum depositional age of modern global sediment. *Earth-Sci. Rev.* **203**, 103109 (2020).

55. Zhu, Z., Campbell, I. H., Allen, C. M., Brocks, J. J. & Chen, B. The temporal distribution of Earth's supermountains and their potential link to the rise of atmospheric oxygen and biological evolution. *Earth Planet. Sci. Lett.* **580**, 117391 (2022).
56. Brown, M. & Johnson, T. Secular change in metamorphism and the onset of global plate tectonics. *Am. Miner.* **103**, 181-196 (2018).
57. Brown, M. & Johnson, T. Metamorphism and the evolution of subduction on Earth. *Am. Miner.* **104**, 1065-1082 (2019).
58. Ferry, J. M. & Watson, E. B. New thermodynamic models and revised calibrations for the Ti-in-zircon and Zr-in-rutile thermometers. *Contrib. Mineral. Petrol.* **154**, 429-437 (2007).
59. Jourdan, F., Nomade, S., Wingate, M. T. D., Eroglu, E. & Deino, A. Ultraprecise age and formation temperature of the Australasian tektites constrained by $^{40}Ar/^{39}Ar$ analyses. *Meteorit. Planet. Sci.* **54**, 2573-2591 (2019).
60. Koppers, A. A. P. ArArCALC—software for $^{40}Ar/^{39}Ar$ age calculations. *Comput. Geosci.* **28**, 605-619 (2002).
61. Feng, Q. et al. Clockwise P-T-t path of Paleoproterozoic metamorphism from the Dengfeng Complex, southern North China Craton. *Precambrian Res.* **381**, 106846 (2022).
62. Wu, C. M. & Chen, H. X. Revised Ti-in-biotite geothermometer for ilmenite- or rutile-bearing crustal metapelites. *Sci. Bull.* **60**, 116-121 (2015).
63. Fu, D. et al. Boninitic blueschists record subduction initiation and subsequent accretion of an arc–forearc in the northeast Proto-Tethys Ocean. *Geology*. **50**, 10-15 (2022).
64. Wu, C. & Chen, H. Calibration of a Ti-in-muscovite geothermometer for ilmenite- and Al_2SiO_5-bearing metapelites. *Lithos*. **212-215**, 122-127 (2015).
65. Cherniak, D. J. Pb diffusion in rutile. *Contrib. Mineral. Petrol.* **139**, 198-207 (2000).
66. Xiang, H. & Connolly, J. A. D. GeoPS: An interactive visual computing tool for thermodynamic modelling of phase equilibria. *J. Metamorph. Geol.* **40**, 243-255 (2022).
67. Holland, T. J. B. & Powell, R. An improved and extended internally consistent thermodynamic dataset for phases of petrological interest, involving a new equation of state for solids. *J. Metamorph. Geol.* **29**, 333-383 (2011).
68. White, R. W., Powell, R., Holland, T. J. B., Johnson, T. E. & Green, E. C. R. New mineral activity-composition relations for thermodynamic calculations in metapelitic systems. *J. Metamorph. Geol.* **32**, 261-286 (2014).
69. Lan, C., Long, X., Zhai, M. & Wang, J. Depositional age and geochemistry of the 2.44–2.32 Ga Granular Iron Formation in the Songshan Group, North China Craton: Tracing the effects of

atmospheric oxygenation on continental weathering and seawater environment. *Precambrian Res.* **357**, 106142 (2021).

70. Zhang, H. et al. Detrital zircon U–Pb, Lu–Hf, and O isotopes of the Wufoshan Group: Implications for episodic crustal growth and reworking of the southern North China craton. *Precambrian Res.* **273**, 112-128 (2016).
71. Meng, Y. et al. The earliest clastic sediments overlying the Xiong'er volcanic rocks: Implications for the Mesoproterozoic tectonics of the southern North China Craton. *Precambrian Res.* **305**, 268-282 (2018).
72. Hu, G. H., Zhao, T. P., Zhou, Y. Y. & Yang, Y. Depositional age and provenance ofthe Wufoshan Group in the southern margin of the North China Craton: evidencefrom detrital zircon U–Pb ages and Hf isotopic compositions. *Geochimica*. **41**, 326-342 (2012).
73. Kohn, M. J. A refined zirconium-in-rutile thermometer. *Am. Mineral*. **105**, 963-971 (2020).
