# Supplementary material for: Changes in orogenic style and surface environment recorded in Paleoproterozoic foreland successions
Source: Nat Commun. 2023 Dec 2;14:7997. doi: 10.1038/s41467-023-43893-w (PMC10693560; doi:10.1038/s41467-023-43893-w)
Supplement: Supplementary file 3 — Description of Additional Supplementary Files [file 41467_2023_43893_MOESM3_ESM.pdf]

<u>Description of Additional Supplementary Files</u>

File name: Supplementary Data 1

Description: Data tables of zircon and rutile U–Pb isotopes and trace elements.

File name: Supplementary Data 2

Description: Mineral compositions and biotite/muscovite Ti temperature calculations of metasedimentary rocks.

File name: Supplementary Data 3

Description: $^{40}Ar/^{39}Ar$ isotopic data of amphibole and white mica of the Dengfeng Complex.

File name: Supplementary Data 4

Description: Compilation of zircon U-Pb isotopic ratios and ages of Songshan Group and Wufoshan Group.
